# Self-Esteem as a Mediator Between Body-Esteem and Depression Among Korean Adolescents: Differences by Weight Status

**DOI:** 10.3390/healthcare14050616

**Published:** 2026-02-28

**Authors:** So-Yeon Kim, Yong-Sook Eo

**Affiliations:** College of Nursing, Dongguk University WISE, Gyeongju 38066, Republic of Korea; smallpond588@naver.com

**Keywords:** adolescent, body image, self-esteem, depression, obesity

## Abstract

**Highlights:**

**What are the main findings?**

- Self-esteem mediated the relationship between body-esteem and depression among adolescents, with different mediation patterns across BMI groups.
- The mediation effect was partial in normal-weight adolescents and full in adolescents who are overweight or obese.

**What are the implications of the main findings?**

- Mental health interventions should be tailored based on weight status, addressing body image and global self-esteem in normal-weight adolescents.
- Programs for adolescents who are overweight or obese should prioritize strengthening self-esteem to reduce vulnerability to depressive symptoms.

**Abstract:**

Background/Objectives: Body-esteem during adolescence is associated with depression, potentially through self-esteem, a key indicator of global self-worth. However, evidence regarding whether this mediating pathway differs by weight status remains limited. This study examined the mediating role of self-esteem in the relationship between body-esteem and depression among normal-weight adolescents and those with overweight or obesity. Methods: This cross-sectional secondary analysis utilized data from 1168 nationally representative 14-year-old adolescents who participated in the 15th wave of the Panel Study on Korean Children (2022). Data were collected between July and December 2022 through home visits conducted by trained interviewers. Mediation analysis was conducted using PROCESS Macro Model 4, adjusting for sociodemographic and psychosocial covariates. Results: Based on BMI classification, 77.7% of participants were normal weight and 22.3% were overweight or obese. Body-esteem was higher in normal-weight adolescents than in those with overweight or obesity. In both groups, body-esteem was positively associated with self-esteem and negatively associated with depression. After covariate adjustment, self-esteem partially mediated the association between body-esteem and depressive symptoms in normal-weight adolescents and fully mediated this association in adolescents with overweight or obesity. Conclusions: The psychological pathways linking body-esteem and depression differed by weight status. Self-esteem mediated this association in both groups, with a stronger mediating role identified among adolescents with overweight or obesity. These findings highlight the importance of considering weight status when examining psychological pathways related to body perception and emotional well-being.

## 1. Introduction

### 1.1. Background

Adolescence is a critical developmental transition phase from childhood to adulthood, characterized by rapid physiological, emotional, and social changes [1]. Pubertal development leads to heightened awareness of physical appearance and increased sensitivity to peer and social evaluations, which can contribute to psychological vulnerability [2]. Emotional instability is common during this period, and depression has become one of the most prevalent mental health issues among adolescents [3]. Depression is characterized by persistent sadness and lack of interest and is associated with emotional, physical, and cognitive changes across multiple domains [3]. These developmental characteristics make adolescents particularly sensitive to influences from their social environment, and in cultural contexts where appearance and body image are highly emphasized, emotional challenges may be further intensified [4,5,6].

In South Korean society, physical appearance and body characteristics are considered crucial for evaluating individuals [4,7], and within the member countries of the Organization for Economic Co-operation and Development (OECD), Koreans are classified as having relatively lean body types [8]. These societal body norms are reflected in adolescents, with Korean adolescents reporting body image- and appearance-related stress as the second most significant source of stress, following academic stress [9]. Such social environments may heighten adolescents’ sensitivity to their appearance and body, potentially increasing their emotional burden and psychological vulnerability [6].

South Korean adolescents reported negative emotions, such as ‘always feeling sad’, at rates approximately four times higher than those observed in Western European countries [10]. Large-scale surveys show that 27.7% of Korean adolescents experience depressive symptoms [11]. Recent data indicate a significant increase in diagnosed depression and anxiety disorders among children and adolescents, with mental health problems contributing substantially to suicide risk, which is the leading cause of adolescent mortality [12]. Identifying its contributing factors is essential for early detection and intervention because adolescent depression is associated with poor academic achievement, maladjustment to school, and impaired peer relationships [1].

Factors influencing adolescent depression include objective indicators such as Body Mass Index (BMI), as well as subjective perceptions and evaluations of body weight and appearance [13]. Previous studies have shown that adolescents with overweight or obesity report higher levels of depressive symptoms than their normal-weight peers [14,15]. However, subjective weight perception was reported to be more strongly associated with depression than actual weight status [16]. These findings highlight the importance of examining body-esteem, defined as an individual’s subjective perception and evaluation of their body, in addition to objective BMI, when investigating adolescent depression.

Body-esteem reflects the overall evaluation of one’s body, encompassing both satisfaction and dissatisfaction [17,18]. During adolescence, body-esteem is shaped by social comparisons, peer feedback, and external evaluations [19]. In South Korean society, physical appearance is a prominent criterion for self-evaluation [4], and approximately 17% of Korean adolescents face discrimination based on appearance, which is associated with a more negative body-esteem [20]. Negative body-esteem has been consistently reported as a significant predictor of mental health problems, including depression [17,21,22].

The relationship between negative body-esteem and depressive symptoms may be influenced by intra-individual psychological factors such as self-esteem and resilience [23]. Consequently, recent research has emphasized the importance of identifying protective factors that may buffer the effect of negative body perceptions on depression [5,17,24]. Self-esteem is defined as the degree to which an individual perceives themselves as valuable and positive [25] and is closely associated with body-esteem [5]. A positive body perception has been shown to improve self-esteem, while a negative body perception diminishes it [17]. Furthermore, higher self-esteem is associated with lower levels of depression and is deemed a robust negative predictor of depressive symptoms [26].

Several studies suggest that self-esteem mediates the relationship between body-esteem and depression instead of serving as a direct correlate [21]. However, findings remain inconsistent regarding whether this mediation is partial or full [21,26,27]. Moreover, prior research indicates that the strength of the mediating effect of self-esteem may vary across cultural contexts [21], suggesting that sociocultural norms surrounding appearance could influence this psychological pathway. Weight status may also influence these pathways. Adolescents with overweight or obesity tend to report lower body satisfaction and self-esteem, as well as higher levels of depression, compared with their normal-weight peers [28]. In sociocultural environments that strongly emphasize appearance-based evaluation, weight-related stigma may intensify negative body perceptions and potentially alter the mediating role of self-esteem in the relationship between body-esteem and depression [21].

Despite these findings, important gaps remain in the literature. First, few studies have directly compared the mediating role of self-esteem across weight-status groups, leaving it unclear whether the pattern of mediation differs between normal-weight adolescents and those with overweight or obesity. Second, many prior studies included broad age ranges, potentially confounding developmental differences in body image and self-concept formation. Given that depressive symptoms peak during mid-adolescence (ages 14–17) [29], and that age 14 marks a critical period of pubertal change and self-concept reconstruction when body-esteem and self-esteem are closely interrelated [30], age-specific investigation is warranted. To address these gaps, the present study focused on a single-age cohort of 14-year-old adolescents to minimize developmental stage variability and to examine whether self-esteem mediates the relationship between body-esteem and depression by comparing normal-weight adolescents with those who are overweight or obese.

### 1.2. Purpose

The purpose of this study was to examine the mediating role of self-esteem on the relationship between body-esteem and depression among normal-weight adolescents and adolescents who were overweight or obese.

The specific objectives were as follows:

1. To assess levels of body-esteem, self-esteem, and depression, and identify differences between normal-weight adolescents and adolescents with overweight or obesity.
2. To examine the correlations among body-esteem, self-esteem, and depression in each weight-status group.
3. To examine whether self-esteem mediates the relationship between body-esteem and depression in normal-weight adolescents and adolescents with overweight or obesity.

## 2. Materials and Methods

### 2.1. Study Design

Using data from the Panel Study on Korean Children (below PSKC), a cross-sectional secondary data analysis was conducted to examine the mediating role of self-esteem on the relationship between body-esteem and depression among adolescents. Although the PKSC is a longitudinal panel, the 15th wave (2022) was selected because variations in key measures and respondents across waves limit longitudinal comparability.

### 2.2. Participants and Ethical Considerations

Data from the 15th Wave of the PSKC, conducted in 2022, is a nationally representative longitudinal cohort of Korean children. The PSKC was initiated in 2008 with 2150 newborns recruited from medical institutions across Korea and has conducted annual follow-ups through 2027 [31]. The current analysis included adolescents aged 14 years. The PSKC collects data from children, parents, and teachers using a multi-stage data collection procedure. Initial contact is conducted via telephone screening, followed by the distribution of paper questionnaires and home visits by trained research staff. During home visits, interviews, observations, and performance-based assessments are conducted using Tablet-Assisted Personal Interview (TAPI) software. Anthropometric measurements (height and weight) were obtained by trained researchers, while psychosocial variables were assessed using standardized self-report questionnaires administered during face-to-face interviews.

**Figure 1:**
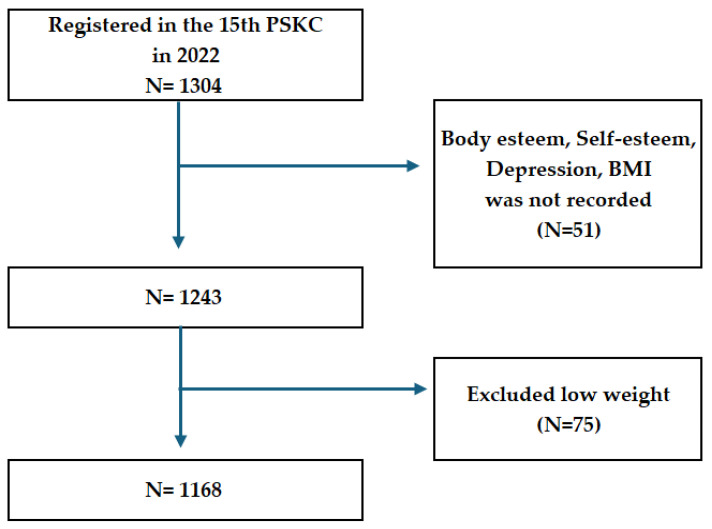
Flow chart of participants.

Among the 1304 adolescents who participated in the 15th wave, 51 with missing data on key variables (body-esteem, self-esteem, depression, and BMI) and 75 classified as underweight were excluded due to the relatively small sample size and concerns regarding statistical stability between groups. Consequently, data from 1168 adolescents were included in the final analysis (Figure 1). The PSKC survey was approved by the Institutional Review Board of the Korea Institute of Child Care and Education (KICCE) (IRB No. KICCEIRB-2022-07). Written informed consent was obtained from all participants and their guardians prior to data collection. The present study is a secondary analysis of de-identified data, and no additional data collection or interventions were conducted.

### 2.3. Measures

#### 2.3.1. Obesity (BMI)

Weight status was determined using the BMI, calculated from the measured height and weight. According to the 2017 Korean National Growth Charts (Korean Pediatric Society), age- and sex- specific BMI percentiles were used to classify weight status as normal weight (5th ≤ percentile < 85th), overweight (85th ≤ percentile < 95th), and obese (≥95th percentile). In this study, adolescents classified as overweight or obese were combined into a single group and compared with a normal-weight group.

#### 2.3.2. Body-Esteem

Body-esteem was assessed using a scale adapted by KICCE from the Body-Esteem Scale developed by Mendelson and White [32]. The instrument consists of five items, including four items assessing appearance satisfaction and one item related to height, which was added after an expert review. Content validity of the adapted scale was established through peer review conducted by KICCE and expert consultation, followed by pilot testing prior to finalization. The items assess adolescents’ global evaluation of their physical appearance and are rated on a 4-point Likert scale, ranging from 1 (strongly disagree) to 4 (strongly agree). Higher scores indicate more positive body-esteem. The Cronbach’s α was 0.70 in a previous study [33] and 0.66 in this study. Cronbach’s alpha values above 0.60 are generally considered acceptable in exploratory research contexts [34].

#### 2.3.3. Self-Esteem

Self-esteem was measured using a five-item shortened version of Rosenberg’s Self-Esteem Scale [25], adapted by KICCE, based on the Millennium Cohort Study Child Paper Self-Completion Questionnaire. This age-appropriate instrument was developed with consideration of children’s cognitive and linguistic comprehension while preserving the core construct of global self-worth [35]. The scale was modified to ensure cultural and developmental appropriateness for Korean children and has been implemented in national panel surveys. The instrument measures adolescents’ perceived self-worth and sense of competence. Items are rated on a 4-point Likert scale ranging from 1 (strongly disagree) to 4 (strongly agree), with higher scores indicating higher levels of self-esteem. The Cronbach’s α was 0.90 in a previous study [36] and 0.87 in this study, indicating good internal consistency.

#### 2.3.4. Depression

Depressive symptoms were assessed using the Korean version of the 11-item Center for Epidemiologic Studies Depression Scale for Children (CES-DC 11) [37], which was abbreviated and validated by Heo, Lee, and Kim [38] from the original 20-item CES-DC developed for adolescents. The construct validity of the CES-DC 11 was supported through confirmatory factor analysis, which verified its factor structure [38]. This self-report instrument measures the frequency of depressive symptoms, including persistent sadness and anhedonia, experienced during the past week. Each item is rated on a 4-point scale ranging from 1 (strongly disagree) to 4 (strongly agree), with higher scores indicating greater severity of depressive symptoms. The Cronbach’s α was 0.91 in previous research [38] and 0.85 in this study.

#### 2.3.5. Covariates

Covariates were selected based on previous studies and included sex, living arrangement, sleep duration, peer attachment, academic stress, family communication, and subjective economic status (SES). Sex was categorized as male or female. Living arrangement was classified as cohabiting with both parents or living with a single parent. Sleep duration was calculated based on the average bedtime and wake-up time on weekdays. Following previous research [39], sleep duration was categorized into three groups: ≤6 h, 7–8 h, and ≥9 h daily. Peer attachment refers to the emotional bonds formed with peers and was assessed using a 4-point Likert scale ranging from 1 (strongly disagree) to 4 (strongly agree). The scale included three subdomains: communication (three items), trust (three items), and alienation (three items), with higher scores indicating stronger levels of each construct. The Cronbach’s α coefficients for the subdomains were 0.73 for communication, 0.78 for trust, and 0.66 for alienation. Academic stress was measured using four items assessing stress associated with schoolwork, rated on a 5-point Likert scale ranging from 1 (never) to 5 (always), with higher scores indicating greater academic stress. The Cronbach’s α for this scale was 0.82 in this study. Family communication was assessed using 10 items evaluating the quality of parent–child communication on a 5-point Likert scale ranging from 1 (never) to 5 (always), with higher scores indicating more positive communication. The Cronbach’s α was 0.93. Subjective economic status was measured using a 10-point ladder scale representing adolescents’ perceived relative socioeconomic position, ranging from 1 (“Very poor”) to 10 (“Very well-off”).

### 2.4. Data Analysis

Statistical analyses were conducted using Statistical Package for the Social Sciences (SPSS) version 29 (IBM Corp., Armonk, NY, USA) and the PROCESS Macro for SPSS (version 3.3, Model 4) [40]. Normality was assessed using skewness and kurtosis values, ranging from −0.376 to 1.471 and 0.232 to 3.236, respectively, and were within acceptable thresholds (|skewness| < 3 and |kurtosis| < 8). Descriptive statistics, including frequencies, percentages, means, and standard deviations, were used to summarize participant characteristics and study variables. Differences between BMI groups were examined using independent t-tests and one-way analysis of variance with Scheffé’s post hoc test. Pearson’s correlation coefficients were calculated to examine the associations between body-esteem, self-esteem, and depression. The mediating role of self-esteem was examined using PROCESS Model 4 with 5000 bootstrap samples and 95% bias-corrected confidence intervals (CIs). The indirect effect was considered statistically significant when the 95% CI did not include zero.

## 3. Results

### 3.1. General Characteristics of Participants

The general characteristics of the participants and differences according to BMI group are presented in Table 1. Among the 1168 adolescents included in the analysis, 907 (77.7%) were classified as normal-weight and 261 (22.3%) as overweight or obese. The sample population comprised 599 males (51.3%) and 569 females (48.7%). A significant difference in gender distribution was observed between BMI groups (χ^2^ = 27.69, *p* < 0.001). Most participants lived with both parents (n = 1039, 89.0%), whereas 129 (11.0%) lived with a single parent. Sleep duration differed significantly between groups (χ^2^ = 6.54, *p* = 0.038), and subjective socioeconomic status (SES) also differed significantly (t = 2.31, *p* = 0.021), with higher scores reported by the normal-weight group (5.40 ± 1.37) than in the overweight/obese group (5.19 ± 1.23). Regarding peer attachment, communication (t = 2.82, *p* = 0.005) and trust (t = 2.67, *p* = 0.008) were significantly higher in the normal-weight group.

### 3.2. Differences in Body-Esteem, Self-Esteem, and Depression by BMI Group

Differences in body-esteem, self-esteem, and depression between BMI groups are shown in Table 2. Body-esteem was significantly higher among normal-weight adolescents (13.51 ± 2.55) than among adolescents who were overweight or obese (13.11 ± 2.64; t = 2.17, *p* = 0.030; Cohen’s *d* = 0.16), although the effect size was small. No statistically significant differences were found between the groups in self-esteem (t = 0.81, *p* = 0.418) or depressive symptoms (t = 0.30, *p* = 0.763).

### 3.3. Differences in Key Variables According to General Characteristics

Table 3 shows the differences in depression according to general characteristics in the normal-weight and overweight/obese groups. Additional results for body-esteem and self-esteem are provided in Appendix A, Table A1. In the normal-weight group, males reported lower depression than females. Sleep duration was significantly associated with depression (F = 6.45, *p* = 0.002), with adolescents sleeping ≤ 6 h per day reporting higher depression.

Peer attachment (communication, trust, and alienation) was significantly associated with depression in both BMI groups. Greater peer communication and trust were associated with lower depression, while peer alienation was associated with higher depression (all *p* < 0.001). Academic stress was positively associated with depression in both BMI groups (all *p* < 0.001). Family communication was negatively associated with depression in both groups (all *p* < 0.001).

### 3.4. Correlations Between Body-Esteem, Self-Esteem, and Depression

Table 4 shows the correlations between body-esteem, self-esteem, and depression in the normal-weight and overweight/obese groups. In the normal-weight group, body-esteem significantly and positively correlated with self-esteem (r = 0.60, *p* < 0.001). Depression significantly and negatively correlated with body-esteem (r = −0.43, *p* < 0.001) and self-esteem (r = −0.43, *p* < 0.001). In the overweight/obese group, body-esteem significantly and positively correlated with self-esteem (r = 0.63, *p* < 0.001). Depression significantly and negatively correlated with body-esteem (r = −0.40, *p* < 0.001) and self-esteem (r = −0.61, *p* < 0.001). The correlation coefficients among the study variables ranged from 0.40 to 0.63, which were considered acceptable and did not indicate problematic multicollinearity (r ≥ 0.70) [41].

### 3.5. Mediated Analysis Between Body-Esteem and Depression via Self-Esteem by BMI Group

**Figure 2:**
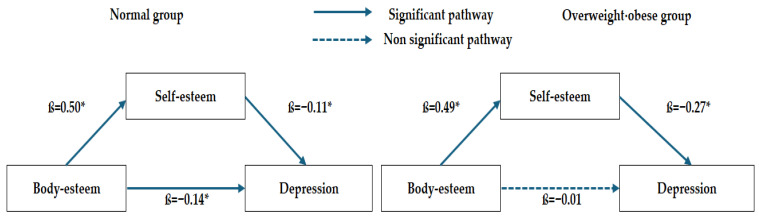
Mediated analysis between body-esteem and depression via self-esteem by BMI group (* *p* < 0.05).

The mediating role of self-esteem in the association between body-esteem and depression by weight status is presented in Table 5 and Figure 2. Mediation analyses were conducted after adjusting for covariates that were significantly associated with depression in the univariate analyses, including sleep duration, peer attachment, academic stress, and family communication. In the normal-weight group, body-esteem had a significant positive effect on self-esteem (ß = 0.50). Body-esteem (ß = −0.14) and self-esteem (ß = −0.11) were significant predictors of depression. The indirect effect of body-esteem on depression through self-esteem was statistically significant (ß = −0.05, 95% CI: −0.09 to −0.02).

In the overweight/obese group, body-esteem significantly predicted self-esteem (ß = 0.49), and self-esteem significantly predicted depression (ß = −0.27). However, the direct effect of body-esteem on depression was not significant (ß = −0.01, *p* = 0.895). The indirect effect of body-esteem on depression through self-esteem remained significant (ß = −0.13, 95% CI: −0.24 to −0.04).

## 4. Discussion

In this study, we examined the differences in the mediating role of self-esteem in the association between body-esteem and depression across different weight status groups. The key findings are interpreted in relation to the previous literature and their implications for adolescent mental health interventions.

First, differences in body-esteem, self-esteem, and depression between normal-weight adolescents and adolescents who were overweight or obese were examined. Body-esteem was significantly higher among normal-weight adolescents than among adolescents who were overweight or obese, consistent with previous studies reporting lower body image satisfaction among adolescents with obesity [24,42]. Previous research has indicated that adolescents’ body-esteem is more strongly influenced by personal perceptions of weight than objective indicators, including BMI [43]. A comparative study of Korean and U.S. adolescents found that Korean adolescents reported higher levels of body dissatisfaction and were more likely to perceive themselves as overweight, regardless of their actual weight status [21]. A national comparative study further reported cross-national differences in body image perception, indicating that adolescents who are overweight or obese are more likely to perceive themselves as overweight and engage in weight-control behaviors, including dieting [44]. These findings indicate that adolescents’ body-esteem may be shaped by objective physical indicators such as BMI and sociocultural factors. In South Korea, where appearance-related social norms and evaluations are highly emphasized [45], adolescents may be more likely to engage in social comparison processes that can affect their body-esteem.

The mean self-esteem score in this study, which was 78.3 (converted to a 100-point scale), was similar to the score Becerra et al. [46] reported among Spanish adolescents aged 12–14 years and higher than the score Kim and Jo [47] reported among Korean school-aged children. Self-esteem fluctuates considerably during the transition from childhood to adolescence and tends to stabilize thereafter [48]. Therefore, differences in self-esteem across studies may reflect age-related developmental variations.

The mean depression score was 16.7 (100-point scale), which was similar to the findings of Song et al. [49] among adolescents aged 10–14 years and lower than those reported by Jeon et al. [50] among adolescents aged 14–16 years. Previous studies have reported that depressive symptoms can increase during the transition from early to mid-adolescence as academic and career-related demands intensify, contributing to a greater emotional burden [29]. Adolescence is characterized by substantial psychological changes [30], and mid-adolescence is a period of heightened vulnerability to depression [29]. In this study, academic stress was positively associated with depressive symptoms, supporting previous evidence that academic pressure majorly contributes to psychological distress among adolescents. The CES-DC 11 scale is widely used to assess depressive symptoms in children and adolescents [38]; however, consensus regarding the clinical cutoff points remains limited. Future studies should establish clinically validated thresholds and examine the diagnostic utility of this scale.

Lower body-esteem and self-esteem were associated with higher levels of depression in both BMI groups. This finding aligns with previous studies reporting that a negative body image is associated with increased depressive and anxiety symptoms during adolescence [17,26]. Notably, the negative correlation between self-esteem and depression was stronger among adolescents who were overweight or obese (r = −0.61) than among normal-weight adolescents (r = −0.43). Adolescents who are overweight or obese are more likely to experience weight-related stigma and negative social experiences, associated with depression, anxiety, and reduced self-esteem [51,52]. These negative social experiences may exacerbate emotional vulnerability by undermining self-esteem [26,52]. These findings show that sociocultural appearance-related pressures and peer experiences may disproportionately affect adolescents who are overweight or obese, contributing to lower self-esteem and greater depressive symptoms. Additionally, the positive association between body-esteem and self-esteem in both groups aligns with previous studies showing that positive body perceptions are associated with higher self-esteem [22,26]. Overall, body-esteem and self-esteem are key psychosocial factors in understanding adolescent depression, especially among adolescents who are overweight or obese, in whom self-esteem may play a more prominent role in emotional vulnerability.

Mediation analyses revealed different pathway structures in the two BMI groups. In normal-weight adolescents, self-esteem partially mediated the relationship between body-esteem and depression, indicating that body-esteem contributes to depression directly and indirectly through self-esteem. This finding aligns with previous research showing a partial mediation of self-esteem in the relationship between body dissatisfaction and depression among Korean adolescents [21] and female university students [26]. The results suggest that, in normal-weight adolescents, self-esteem may be maintained through multiple domains, including academic achievement and peer relationships. Accordingly, the adverse impact of negative body-related perceptions may influence depressive symptoms not only through diminished self-esteem but also through additional psychosocial mechanisms.

In contrast, self-esteem fully mediated the relationship between body-esteem and depression among adolescents who are overweight or obese. This indicates that body-esteem influenced depression only indirectly through self-esteem. Similar findings were reported by Kwon et al. [53], who found that body appearance evaluation influenced depression through self-esteem among children with obesity, whereas other studies showed that body dissatisfaction affects depression indirectly through general self-esteem rather than directly [54]. Self-esteem is closely related to individuals’ beliefs, evaluations, and confidence [25] and is an important factor in emotional resilience during childhood and adolescence [54]. Because global self-worth is partially shaped by body image perceptions [55], cultural context may further influence this developmental process [21]. Comparative research between Korean and U.S. adolescents has shown that Korean adolescents, within a more interdependent sociocultural framework, may develop self-esteem in closer relation to perceived social evaluation and relational experiences, whereas U.S. adolescents may internalize body image within a more independent self-construal [21]. In South Korea, where appearance-related norms are salient and social evaluation is emphasized [45], adolescents with overweight or obesity may be particularly vulnerable to negative self-appraisals shaped by perceived societal standards. These findings highlight the importance of self-esteem–enhancing interventions to prevent depression in overweight and obese adolescents. In contrast, for normal-weight adolescents, depressive symptoms may arise through multiple pathways beyond self-esteem; therefore, comprehensive interventions that address peer relationships, academic stress, and broader social evaluative contexts may be warranted.

This study has some limitations that should be considered when interpreting the findings. First, this study used cross-sectional data collected at a single time point in 2022, which limits the ability to establish temporal ordering or draw causal inferences among body-esteem, self-esteem, and depression and precludes the determination of the directionality of these relationships. Although the Panel Study on Korean Children is longitudinal in design, inconsistencies in the measurement of key variables across waves and differences in respondents (child vs. parent reports) limited the feasibility of longitudinal analyses. Second, although internal consistency coefficients were within acceptable ranges, some constructs were assessed using abbreviated versions of original instruments, which may affect construct validity. Further psychometric validation of these shortened measures is recommended. Additionally, although correlations among primary variables were moderate (r = 0.40–0.63) and did not exceed conventional thresholds for multicollinearity, the use of self-report measures collected at a single time point raises the possibility of common method bias and inflated associations. Third, BMI was used to classify weight status; however, BMI does not fully capture body fat distribution or central adiposity. Future studies should consider incorporating alternative anthropometric indices, such as the Body Roundness Index (BRI) or the Body Shape Index (ABSI), to more comprehensively assess body composition. Finally, the underweight group was excluded due to its relatively small sample size and concerns regarding statistical stability, limiting the generalizability of findings to this population. Future research using longitudinal designs with consistent measurement across waves and including diverse weight-status groups is needed to further elucidate these associations.

## 5. Conclusions

The mediating role of self-esteem in the relationship between body-esteem and depression among normal-weight adolescents and adolescents who were overweight or obese was examined in this study. Among normal-weight adolescents, body-esteem was associated with depression directly and indirectly through self-esteem. In contrast, among adolescents with overweight or obesity, body-esteem was associated with depression only indirectly through self-esteem, suggesting a pattern consistent with full mediation. These findings suggest that the psychological pathways linking body-esteem, self-esteem, and depression vary according to weight status. Among normal-weight adolescents, negative body perceptions may be associated with depressive symptoms through multiple mechanisms, highlighting the relevance of interventions that promote positive body image and strengthen self-esteem alongside other internal and contextual resources. In contrast, among adolescents with overweight or obesity, global self-esteem appears to play a more prominent role in emotional adjustment, suggesting that interventions focused on enhancing overall self-worth and fostering stable, positive self-evaluations may be particularly relevant. These results further underscore the importance of developing differentiated, weight status–sensitive mental health strategies to better address depressive symptoms among adolescents.

## Figures and Tables

**Table 1:** Differences in general characteristics of participants according to BMI group (N = 1168).

Characteristics	Categories	n (%) or M ± SD	Normal	Overweight & Obese	χ^2^ or t (*p*)
			(n = 907)	(n = 261)	
			n (%)	n (%)	
Sex	Male	599 (51.3)	430 (47.4)	169 (64.8)	24.40 (<0.001)
	Female	569 (48.7)	477 (52.6)	92 (35.2)	
Living arrangement	Both parents	1039 (89.0)	815 (89.9)	224 (85.8)	3.36 (0.067)
	Single parent	129 (11.0)	92 (10.1)	37 (14.2)	
Sleep duration (hour)	≤6	297 (25.4)	246 (27.1)	51 (19.5)	6.54 (0.038)
	7–8	735 (62.9)	555 (61.2)	180 (69.0)	
	≥9	136 (11.6)	106 (11.7)	30 (11.5)	
Subjective socioeconomic status		5.35 ± 1.34	5.40 ± 1.37	5.19 ± 1.23	2.31 (0.021)
Peer Attachment	Communication	3.16 ± 0.52	3.18 ± 0.51	3.08 ± 0.53	2.82 (0.005)
	Trust	3.17 ± 0.59	3.19 ± 0.58	3.08 ± 0.59	2.67 (0.008)
	Alienation	1.86 ± 0.63	1.86 ± 0.63	1.83 ± 0.61	0.82 (0.415)
Academic stress		2.66 ± 0.88	2.68 ± 0.88	2.57 ± 0.88	1.72 (0.086)
Family Communication		3.92 ± 0.67	3.93 ± 0.67	3.89 ± 0.67	0.72 (0.470)

M = mean; SD = standard deviation.

**Table 2:** Differences in body-esteem, self-esteem, and depression by BMI group (N = 1168).

Variables	Total Mean ± SD	Normal (n = 907) Mean ± SD	Overweight & Obese (n = 261) Mean ± SD	t/(*p*)	Min–Max
Body-esteem	13.42 ± 2.58	13.51 ± 2.55	13.11 ± 2.64	2.17 (0.030)	5–20
Self-esteem	15.66 ± 2.70	15.69 ± 2.64	15.54 ± 2.89	0.78 (0.433)	5–20
Depression	5.52 ± 4.63	5.54 ± 4.57	5.45 ± 4.87	0.29 (0.770)	0–33

SD = standard deviation.

**Table 3:** Differences in depression by general characteristics (N = 1168).

Characteristics	Categories	Depression			
		Normal (n = 907)		Overweight & Obese (n = 261)	
		M ± SD	t/F or r (*p*)	M ± SD	t/F or r (*p*)
Sex	Male	0.48 ± 0.42	−1.70 (0.089)	0.46 ± 0.38	−1.62 (0.107)
	Female	0.53 ± 0.41		0.56 ± 0.53	
Living arrangement	Both parents	0.50 ± 0.41	0.05 (0.961)	0.50 ± 0.41	0.17 (0.868)
	Single parent	0.50 ± 0.44		0.48 ± 0.62	
Sleep duration (hour)	<6 ^a^	0.58 ± 0.44	6.45 (0.002)	0.59 ± 0.47	1.43 (0.241)
	7~8 ^b^	0.47 ± 0.40	b, c < a	0.47 ± 0.44	
	>8 ^c^	0.47 ± 0.40		0.49 ± 0.44	
Subjective socioeconomic status			−0.02 (0.580)		0.08 (0.182)
Peer Attachment	Communication		−0.21 (<0.001)		−0.44 (<0.001)
	Trust		−0.24 (<0.001)		−0.44 (<0.001)
	Alienation		0.24 (<0.001)		0.39 (<0.001)
Academic stress			0.42 (<0.001)		0.33 (<0.001)
Family Communication			−0.39 (<0.001)		−0.47 (<0.001)

M = mean; SD = standard deviation; ^a^, ^b^, ^c^ groups are not significantly different from each other (Scheffé test).

**Table 4:** Correlations among body-esteem, self-esteem, and depression by weight status.

Variables	1	2	3
1. Body-Esteem	-	0.60 ***	−0.43 ***
2. Self-esteem	0.63 ***	-	−0.43 ***
3. Depression	−0.40 ***	−0.61 ***	-

Note. Correlations above the diagonal represent normal-weight adolescents (n = 907); correlations below the diagonal represent adolescents with overweight/obesity (n = 261); *** *p* < 0.001.

**Table 5:** Mediated analysis between body-esteem and depression via self-esteem by BMI group (N = 1168).

Variables		Normal (n = 907)						
		ß	SE	t (*p*)	Adj R^2^	F (*p*)	LLCI	ULCI
Self-esteem	Body-esteem	0.50	0.03	17.74	0.487	106.41	0.44	0.55
				(<0.001)		(<0.001)		
Depression	Body-esteem	−0.14	0.03	−4.74	0.352	54.09	−0.19	−0.08
				(<0.001)		(<0.001)		
	Self-esteem	−0.11	0.03	−3.62			−0.16	−0.05
				(<0.001)				
		Overweight & Obese (n = 261)						
		ß	SE	t (*p*)	Adj R^2^	F (*p*)	LLCI	ULCI
Self-esteem	Body-esteem	0.49	0.05	9.30	0.574	57.00	0.39	0.60
				(<0.001)		(<0.001)		
Depression	Body-esteem	−0.01	0.05	−0.13	0.470	32.10	−0.11	0.10
				(0.895)		(<0.001)		
	Self-esteem	−0.27	0.05	−4.99			−0.37	−0.16
				(<0.001)				

SE = standard error; LLCI = lower limit confidence interval; ULCI = upper limit confidence interval; Adj R^2^ = adjusted R-square. Note: Values were controlled for covariates.

## Data Availability

Data are available upon request owing to ethical and privacy restrictions.

## Appendix A

**Table A1:** Differences in body-esteem and self-esteem by general characteristics (N = 1168).

Characteristics	Categories	Body-Esteem				Self-Esteem			
		Normal (n = 907)		Overweight & Obese (n = 261)		Normal (n = 907)		Overweight & Obesity (n = 261)	
		M ± SD	t/F or r (*p*)	M ± SD	t/F or r (*p*)	M ± SD	t/F or r (*p*)	M ± SD	t/F or r (*p*)
Sex	Male	2.83 ± 0.50	7.69	2.71 ± 0.50	3.64	3.20 ± 0.53	3.64	3.19 ± 0.54	2.99
	Female	2.58 ± 0.49	(<0.001)	2.47 ± 0.55	(<0.001)	3.08 ± 0.52	(<0.001)	2.97 ± 0.61	(0.003)
Living arrangement	Both parents	2.70 ± 0.51	−0.49	2.63 ± 0.51	0.82	3.13 ± 0.53	−0.61	3.10 ± 0.56	−0.61
	Single parent	2.73 ± 0.51	(0.625)	2.56 ± 0.62	(0.412)	3.17 ± 0.52	(0.544)	3.16 ± 0.69	(0.540)
Sleep during (hour)	<6 ^a^	2.57 ± 0.48	10.88	2.56 ± 0.62	0.49	3.08 ± 0.52	2.46	3.00 ± 0.68	1.54
	7~8 ^b^	2.74 ± 0.52	(<0.001)	2.63 ± 0.49	(0.615)	3.16 ± 0.54	(0.086)	3.15 ± 0.57	(0.216)
	>8 ^c^	2.78 ± 0.49	^a^ < ^b^, ^c^	2.67 ± 0.58		3.18 ± 0.49		3.05 ± 0.39	
Subjective socioeconomic status			−0.04		0.02		0.01		−0.07
			(0.265)		−0.735		(0.850)		(0.256)
Peer Attachment	Communication		0.15		0.28		0.34		0.49
			(<0.001)		(<0.001)		(<0.001)		(<0.001)
	Trust		0.2		0.27		0.37		0.47
			(<0.001)		(<0.001)		(<0.001)		(<0.001)
	Alienation		−0.12		−0.18		−0.19		−0.31
			(0.003)		(0.003)		(<0.001)		(<0.001)
Academic stress			−0.39		−0.45		−0.30		−0.37
			(<0.001)		(<0.001)		(<0.001)		(<0.001)
Family Communication			0.3		0.25		0.46		0.48
			(<0.001)		(<0.001)		(<0.001)		(<0.001)

M = mean; SD = standard deviation; ^a, b, c^ groups are not significantly different from each other (Scheffé test).
